# Vertigoheel improves central vestibular compensation after unilateral peripheral vestibulopathy in rats

**DOI:** 10.3389/fneur.2022.969047

**Published:** 2022-09-23

**Authors:** Bérénice Hatat, Romain Boularand, Claire Bringuier, Nicolas Chanut, Stéphane Besnard, Andrea M. Mueller, Kathrin Weyer, Bernd Seilheimer, Brahim Tighilet, Christian Chabbert

**Affiliations:** ^1^Vertidiag, Montpellier, France; ^2^Aix Marseille Université-CNRS, Laboratoire de Neurosciences Cognitives, LNC UMR 7291, Marseille, France; ^3^Unité GDR2074 CNRS, Marseille, France; ^4^Heel GmbH, Baden-Baden, Germany

**Keywords:** vestibular disorders, unilateral vestibular lesion, sequential bilateral lesion, central vestibular compensation, spatial cognitive deficits, natural drug, multitarget medication, Vertigoheel

## Abstract

The aim of this study was to assess the effect of Vertigoheel on central vestibular compensation and cognitive deficits in rats subjected to peripheral vestibular loss. Young adult male Long Evans rats were subjected to bilateral vestibular insults through irreversible sequential ototoxic destructions of the vestibular sensory organs. Vestibular syndrome characteristics were monitored at several time points over days and weeks following the sequential insults, using a combination of behavioral assessment paradigms allowing appreciation of patterns of change in static and dynamic deficits, together with spatial navigation, learning, and memory processes. Vertigoheel administered intraperitoneally significantly improved maximum body velocity and not moving time relative to its vehicle control on days 2 and 3 and on day 2, respectively, after unilateral vestibular lesion (UVL). It also significantly improved postural control relative to its vehicle 1 day after UVL. Conversely, Vertigoheel did not display any significant effect vs. vehicle on the severity of the syndrome, nor on the time course of other examined parameters, such as distance moved, mean body velocity, meander, and rearing. Spatial cognition testing using Y- and T-maze and eight-radial arm maze did not show any statistically significant difference between Vertigoheel and vehicle groups. However, Vertigoheel potentially enhanced the speed of learning in sham animals. Evaluating Vertigoheel's effect on thigmotaxis during the open-field video tracking test revealed no significant difference between Vertigoheel and its vehicle control groups suggesting that Vertigoheel does not seem to induce sedative or anxiolytic effects that could negatively affect vestibular and memory function. Present observations reveal that Vertigoheel improves central vestibular compensation following the unilateral peripheral vestibular loss as demonstrated by improvement of specific symptoms.

## Introduction

The acute vestibular syndrome evoked upon unilateral peripheral vestibulopathy is composed of both static and dynamic symptoms. Static signs include the ocular motor (spontaneous nystagmus) and postural (head tilt, postural instability, and falls while standing erect) deficits that are compensated within a few days or weeks. Dynamic signs include alterations of the vestibulo-ocular and vestibulo-spinal reflexes and locomotion. These dynamic signs are much less compensated or over a longer time (1, 2). The static deficits result from the spontaneous resting activity imbalance between bilateral vestibular nuclei complexes (VNCs), and compensation approximately coincides with the restoration of balanced electrical activity between the VNCs. These events were previously confirmed electrophysiologically in the alert guinea pig (3) and cat (4). By contrast, the compensation of dynamic signs seems independent of a rebalanced activity in the VNCs and is attributed to a more global reorganization of the central nervous system [reviewed in (5–7)].

Bilateral vestibulopathy can lead to spatial cognitive impairment in animals and humans (e.g., impaired spatial working memory, reference memory, and spatial navigation). Although the underlying causes have not so far been fully elucidated, bilateral atrophy of the hippocampus has been reported in humans (8). In animals, the loss of bilateral vestibular inputs evokes a decrease in the length of basal dendrites in the hippocampal CA1 area (9), a downregulation of the M1 muscarinic acetylcholine receptor in all regions of the hippocampus and striatum (10), and abnormal place cell responses and theta rhythm in the hippocampus and entorhinal cortex (9).

Vertigoheel (also referred as VH-04) is a multicomponent, multitarget drug made from natural ingredients for which significant benefits in reducing the frequency, duration, and intensity of vertigo in actively treated patients were previously reported (11–13). These effects may be attributed to the pharmacological effect of the preparation on blood microcirculation (14, 15). Vertigoheel has also revealed significant neurophysiological effects in the rat brain, with preliminary observations suggesting cognition-enhancing properties (16). This study was designed with the aim of assessing the effect of Vertigoheel on central vestibular compensation and spatial cognition after sequential ototoxic vestibular lesion. We used a previously validated model of sequential transtympanic injection of arsanilic acid to induce subsequent loss of peripheral vestibular inputs (17). This model reproduces both the characteristics and time course of the peripheral vestibulopathy encountered in vestibular neuritis and labyrinthitis in humans (18), as well as some aspects of Meniere's disease's late stages (19).

**Figure 1:**
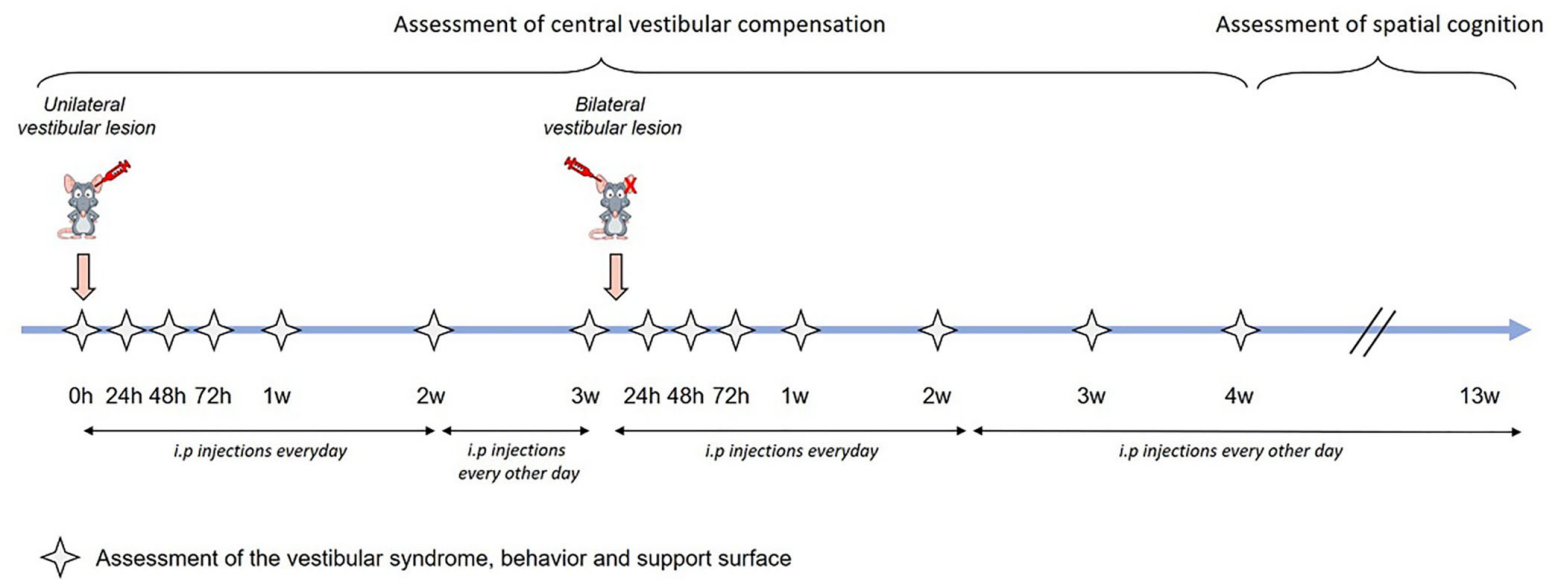
Study timeline. Assessment of central vestibular compensation was made through the analysis of the vestibular syndrome, the behavior and support surface. Each analysis was carried out at several time points: before the transtympanic injection (referred as pre-op), 24 h, 48 h, 72 h, 1 week, 2 weeks, and 3 weeks after unilateral transtympanic injection, and 24 h, 48 h, 72 h, 1 week, 2 weeks, 3 weeks, and 4 weeks after sequential bilateral transtympanic injection. Assessment of spatial cognition started 4 weeks after sequential bilateral transtympanic injection.

**Figure 2:**
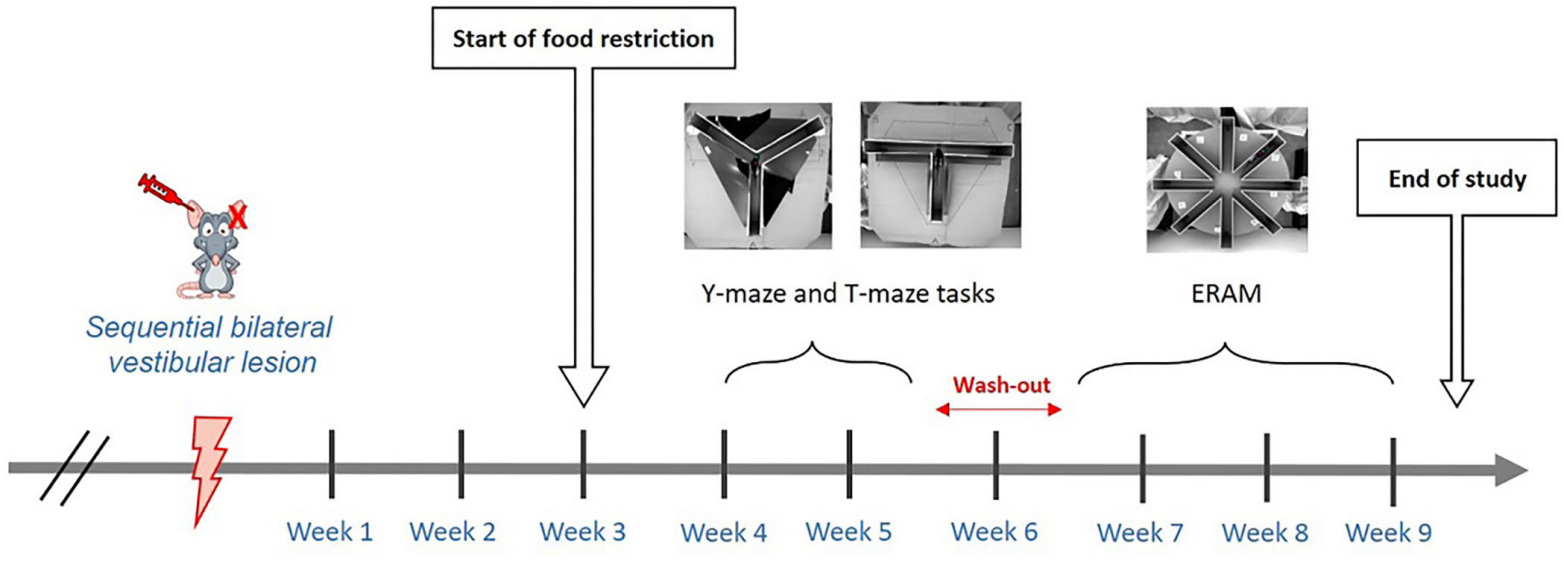
Timeline of the spatial cognition assessment. Assessment of spatial cognition was performed through three sequential behavioral tests: Y-maze, T-maze, and ERAM. Y-maze and T-maze started 4 weeks after sequential bilateral transtympanic injection, whereas ERAM started seven weeks after sequential bilateral transtympanic injection.

Assessment of central vestibular compensation was performed through scoring of the vestibular syndrome, behavior monitored during the open-field test, and support surface in adult rats subjected to sequential transtympanic administration of arsanilic acid (TTA) as previously detailed (17, 20–22). Each analysis was carried out before TTA (referred to as pre-op), and at several time points after both unilateral and bilateral TTA (see study timeline—Figure 1). Assessment of spatial cognition was performed through three sequential behavioral tests: Y-maze, T-maze, and eight-radial arm maze (ERAM). Y-maze and T-maze started 4 weeks after sequential bilateral TTA, whereas ERAM started 7 weeks after sequential bilateral TTA (see study timeline of spatial cognition assessment—Figure 2). The pharmacological effect of Vertigoheel on central vestibular compensation and spatial cognition is discussed. We also studied the potential effect of Vertigoheel on thigmotaxis for several reasons. First, patients who suffer from a vestibular deficiency or dysfunction present a vestibular syndrome, which is also associated with anxiety symptoms. These anxiety symptoms indicate that vestibular function and emotional processes interact together (23). Second, to learn more about the mode of action of Vertigoheel's effect on postural control. Three potential mechanisms could explain the beneficial effect of Vertigoheel on postural control: anxiolysis, cognition enhancement, and modulation of neuronal excitability. The hypothesis of an anxiolytic effect was further analyzed on data collected from the open field.

## Materials and methods

### Animals

All experiments are performed in accordance with the National Institutes of Health's Guide for Care and Use of Laboratory Animals (NIH Publication no. 80-23) revised in 1996 for the UK Animals (Scientific Procedures) Act of 1986 and associated guidelines or the Policy on Ethics approved by the Society for Neuroscience in November 1989 and amended in November 1993 and under the veterinary and National Ethical Committee supervision (French Ministry of Agriculture and Food authorization no. A34169002). The present study was specifically approved by Ethic Committee n°036 from the French National Committee of animal experimentation. Male Long Evans rats (7–8 weeks, Janvier, France) were housed in groups of two under constant temperature (20 ± 2°C), humidity (55 ± 5%), and brightness conditions (lower than 110 Lux). Rats were housed under a 12 h−12 h diurnal light variation (lights on from 07:00 to 19:00 h) with food and water freely available. Handling (~5 min per animal per day) was made 1 week before the beginning of the tests.

### Study medication

VH-04 injection solution and the vehicle control (veh) were manufactured and bottled in 1.1 mL glass ampoules by Heel GmbH (Baden-Baden, Germany) according to the international Good Manufacturing Practice (GMP) standards. The ingredients of VH-04 are listed in Table 1. Each ampoule of the vehicle control contained 0.9% sodium chloride for injection. The study medication was packaged, shipped, and labeled by Heel GmbH, Germany.

**Table 1:** Composition of vertigoheel (VH-04) solution for injection.

**Component**		**Manufacturing method (Ph. Eur.)**	**Initial potency of the ingredient entering the medicinal product**	**Amount of extract (of the plant ingredient) or raw material (other substances) expressed as μg per 1 ml**
*Plant extracts*				
	*Anamirta cocculus*	Method 1.1.8	3	70
	Dried, ripe fruit of *Anamirta cocculus* (L.) Wight and Arn. (syn. *A. paniculata* Colebr.)			
	*Conium maculatum*	Method 1.1.3	2	20
	Fresh, aerial parts of flowering, but not yet fruiting plants of *Conium maculatum* L.			
*Substances of mineral origin*				
	Petroleum rectificatum, petroleum spirit distilling between 180°C and 220°C obtained by rectification of crude oil.	Method 3.1.1	7	1E-04
*Substances of biological origin*				
	*Ambra grisea*	Method monograph specific^*^	5	1.00E-02
	Substance excreted from the intestines of the sperm whale, *Physeter catodon* L. (syn. *Physeter macrocephalus* L.)			

Ph. Eur. = European Pharmacopeia.

* Method equivalent to 2.1.1 Ph Eur.

### Study design

Two groups, Sham-veh (*n* = 16) and Sham-VH-04 (*n* = 16), received sequential bilateral transtympanic injections of 0.9% saline solution and daily i.p. injections of vehicle control (2 mL/kg) and Vertigoheel (2 mL/kg), respectively, during the first 2 weeks following each lesion and every other day until the end of the experiment. Two other groups, TTA-veh (*n* = 15) and TTA-VH-04 (*n* = 15), received sequential bilateral transtympanic injections of arsanilic acid and daily i.p. injections of vehicle control (2 mL/kg) and Vertigoheel (2 mL/kg), respectively, during the first 2 weeks following each lesion and every other day until the end of the experiment. I.p. injections were all made in the morning before experiments. When surgery was planned the same day (unilateral or bilateral), i.p. injections were made during the surgery when animals were under anesthesia. The time between injection and behavioral evaluation varied between 30 min and 3 h. One animal swallowed arsanilic acid *via* the Eustachian tube during the unilateral vestibular lesion and then displayed symptoms of severe respiratory problems. Another animal subjected to unilateral vestibular lesion did not show any symptoms of vestibular disorder. These two animals were excluded from this study.

Assessment of central vestibular compensation was made through scoring of the vestibular syndrome, behavior monitored during the open-field test and the support surface. Each analysis was carried out at several time points before TTA, after UVL, and after bilateral vestibular lesion (BVL). Assessment of spatial cognition was performed through three sequential behavioral tests: Y-maze, T-maze, and ERAM starting 4 weeks after sequential BVL (Figures 1, 2).

### Arsanilic acid lesioning procedure

Each rat received a first single unilateral dose (50 mg/mL to 0.1 mL per ear) of arsanilic acid (Sigma-Aldrich—CAS: 98-50-0) dissolved in 0.9% saline solution in the left ear. Animals were under volatile anesthesia [2% isoflurane in oxygen (flow rate of 2 L/min)]. Arsanilic acid was injected through the anterior part of the tympanum using a 1-mL syringe (needle diameter, 0.8 mm) and arsanilic acid was deposited into the middle-left ear cavity. Three weeks after the first lesion, animals received the sequential bilateral transtympanic injection of arsanilic acid in the right ear. The sham groups received first a unilateral transtympanic injection of 0.9% saline solution (0.1 mL) in the left ear and 3 weeks after the sequential bilateral transtympanic injection of 0.9% saline solution (0.1 mL) in the right ear through the same route.

### Assessment of central vestibular compensation

#### Scoring of vestibular syndrome

Unilateral and bilateral syndromes were quantified at different time points: 24 h, 48 h, 72 h, 1 week, 2 weeks, and 3 weeks after the unilateral lesion and 24 h, 48 h, 72 h, 1 week, 2 weeks, 3 weeks, and 4 weeks after the sequential bilateral lesion. The vestibular syndrome was evaluated using a previously published vestibular scale used for different vestibular insults (24). This scale is based on five static or dynamic locomotor behaviors being together present in the acute phase and sequentially disappearing following a specific time course: (1) *Tumbling* describes spontaneous rotations of the animals along their body axis. It only evokes upon most severe vestibular impairments, appears first following vestibular insults, and lasts several hours (rated 5 on our evaluation scale). (2) *Retropulsion* characterizes the backward movements of the animals. This parameter (rated 4) appears as soon as the rat is able to stand on its four legs and walk again and vanishes after a few days. (3) *Circling* (rated 3) is defined as a spontaneous, stereotypical locomotor activity of movements of the rats in the horizontal plane that starts with the retropulsion behavior but lasts longer, over weeks. (4) *Bobbing* is a spontaneous and intermittent extreme backward extension of the neck leading to abnormal head movements. This behavior (rated 2) is observed over weeks. (5) *Head tilt* (rated 1) is seen in vestibulo-lesioned rats where the head tilts to the side and follows the whole duration of the vestibular syndrome and accompanies even slight and reversible vestibular insults. It is related to an asymmetrical change in muscular tension in the neck. Zero is given when none of these behaviors is observed. In this study, two kinds of scoring have been carried out. The first one is based on the *severity of the whole syndrome*. We distinguished five states from the most severe to the least. A first state in which all symptoms are expressed (rated 15), followed by a second stage in which the tumbling has gone (rated 10). A third state in which both the tumbling and the retropulsion behaviors were absent was rated 6. The following state in which tumbling, retropulsion, and circling were gone was rated 3. Then, two states (rated 2 and 1 respectively) related to states in which both bobbing and head tilt, or head tilt alone remained. In parallel, we have developed a second vestibular scale based on the *severity of each symptom*, which were themselves scored from zero to three. Zero was given when the symptom was not observed. When the symptom was not clearly observed but underlying it was rated 1. Two was given when the symptom was clearly observed, and three was given when the symptom was observed at a maximum degree.

#### Support surface

The support surface is a sensitive parameter used for evaluating vestibular lesion-induced static posture deficit and recovery by measuring the surface delimited by the four paws of the animal after a tail hanging landing test. This test consisted in taking the animal by the tail and lifting it vertically over a height of about 50 cm (lift duration 2 s; position holding at upper position: 1 s). To quantify the support surface, animals were placed in a device with a graduated transparent floor that allowed them to be filmed from underneath. A scale drawn on the bottom served to take measurements of the four paws' location. When the animal landed after the tail hanging test and touched the ground, we captured the four paws' location. Between 10 and 15 measurements were taken for each rat at each time point during recovery (24 h, 48 h, 72 h, 1 week, 2 weeks, and 3 weeks after the unilateral lesion, and 24 h, 48 h, 72 h, 1 week, 2 weeks, 3 weeks, and 4 weeks after the sequential bilateral lesion). An average was calculated for each time point. The support surface was measured using an image analysis system called GNU Octave.

#### Open field and video tracking

The open field apparatus was an inescapable square area (80 × 80 × 40 cm) without bedding litter. Animals were placed in the center of the field considered more anxiogenic compared to the periphery and their behavior was recorded for 10 min using a digital camera with the Ethovision^TM^ XT 15 software (Noldus), which automatically detected the following body points throughout the recordings: nose point, center point, and tail base. This test was carried out before the unilateral lesion (time point called pre-op), 24 h, 48 h, 72 h, 1 week, 2 weeks, and 3 weeks after the unilateral lesion and 24 h, 48 h, 72 h, 1 week, 2 weeks, 3 weeks, and 4 weeks after the sequential bilateral lesion. The day before the pre-op recording, each animal was allowed to freely explore the maze for 2 min. The light was fixed at 40–45 Lux at the center and 30–35 Lux in each corner of the maze. The surfaces of the open field were cleaned thoroughly between animals with an Ethanol solution (20%). We used one profile for five variables (distance moved, mean body velocity, max. body velocity, not moving time, and meander) that we selected for analysis. This profile, a tool included in EthoVision^TM^ XT software, uses the minimal distance moved (MDM) smoothing method to filter out small movements (<0.7 cm) of the animal's center point that are caused by random noise. “Duration not moving” of animals was calculated with an average interval of three samples, one sample is generated every 0.04 s during the whole time of the video, and a threshold of 2.00 cm/s for start and 1.75 cm/s for stop velocity. Mean body velocity and max. body velocity were calculated with an average of three samples. Rearing was manually calculated by an observer during the 10-min session of the open-field test. For the analysis of thigmotaxis, the arena was divided into three concentric zones for analysis: an outer square (80 × 80 cm), an intermediate square (53 × 53 cm), and an inner square (27 × 27 cm). The three parameters (distance moved, cumulative duration, and frequency) were automatically calculated by the Ethovision^TM^ XT 15 software. Frequency was defined as the number of entries in the considered zone. An entry is counted in one zone when the center point has moved to this zone. The frequency used for the zone transitions is calculated unidirectionally from the intermediate zone to the central or the outer zones.

### Assessment of spatial cognition

The assessment of spatial cognition started 4 weeks after the sequential bilateral transtympanic injection (see Figure 2). The first test performed was the Y-maze followed by the T-maze. When the T-maze was stopped, animals were at rest without any test for 1 week. Then, the habituation phase of the ERAM started, followed by the training phase.

#### Spontaneous alternation test (Y-maze)

Immediate spatial memory was assessed by recording spontaneous alternation in a single session in a Y-maze. The maze consisted of three equally spaced arms (50 × 10 × 30 cm). Each animal, naïve to the maze, was placed at the end of one arm randomly determined and allowed to freely explore the maze during a 5-min session. The light was fixed at 40–45 Lux at the center and 25–30 Lux at the end of each arm. A wide-angle video camera was placed 1 m above the center of the maze, allowing real-time monitoring and recording. The number and the sequence of arm entries were collected by an observer. An arm entry was scored when all four paws crossed into the arm. The percentage of alternation was calculated by the formula: (number of alternations/[total number of arm entries − 2]) × 100, where the alternation was defined as consecutive entries into three different arms. This measure is considered to reflect working short-term memory in rodents due to their innate exploratory nature. Additionally, the number of arm entries was calculated as an index of locomotor activity (20). In this study, this test was performed 4 weeks after the sequential bilateral transtympanic injection.

#### Reverse T-maze

Rats were subjected to a T-maze paradigm to test whether vestibular lesions cause a shift in the learning mechanism and cognitive strategy in finding a food reward. The apparatus was located at the center of a room raised by 30 cm with external cues in front of the left and right arms (a black triangle and a blue square). The T-maze task consisted of three arms, 50 cm in length, 30 cm in height, 10 cm in width, and diverged at a 90° angle from one another. The light was fixed at 45–50 Lux at the center and 25–30 Lux at the end of each arm. A wide-angle video camera was placed 1 m above the center of the maze, allowing real-time monitoring and recording. Animals had restricted food intake 1 week before the beginning of the test to keep their body weight at 90% of their initial weight during the entire duration of the T-maze experiment to induce a motivation for food seeking. The habituation phase consisted of two trials. The first trial consisted of a 10-min session with two animals at the same time in which rewards were placed all over the maze (all along the arms and at the end of each arm). The second trial consisted of a 5-min session for each animal in which rewards were also placed all over the maze. The training phase consisted of six successive trials per day. Food rewards were placed randomly at the distal part of either the left or right arm relative to the starting arm. The location of the food reward remained the same for each rat throughout the trials. When rats visited one of the two accessible arms (all four paws in one test arm), the non-visited arm was then closed to let rats consume or not the reward and go back to the start point and then reinforce the memory. The animal was again allowed to explore the maze after an inter-trial interval (30 s) during which the maze was cleaned and the same arm re-baited. A visit to the baited arm was recorded as a correct entry. Animals were trained at least for 5 days or until the group reached an average performance of up to 75–80% correct responses per day. During the reversal training, a rotation angle of 180° of the T-maze in the room was done so that the baited arm was located at the same place relative to the training phase. The reversal training consisted of six trials, and two parameters were scored: (1) the choice of the arm during the first trial, and (2) the percentage of correct responses obtained for the six trials of the reversal training session. If the animal kept turning toward the same side as in the training phase and did not find the reward, it would be assumed that it used an egocentric strategy from body cues: “response strategy”. If the animal turned toward the same external cue as in the training phase and found the reward, it meant that the animal used an allocentric strategy with a “spatial strategy” (21). Only animals that made one error or less the day before the reversal training were included in the graph describing the strategy used. In this study, the T-maze was performed four weeks after the sequential bilateral transtympanic injection (1 day after the Y-maze session).

#### Eight-radial arm maze (ERAM)

The ERAM test assesses the long-term and working memory of an animal. The apparatus consisted of a circular central platform (diameter: 28.7 cm), and eight identical arms (length: 50 cm) situated 30 cm above the floor with three external cues located on each wall (the fourth wall was in fact a curtain which itself constitutes a visual cue): aluminum cross, colored egg boxes, and colorful pillowcase. The adjacent arms were separated by an angle of 45°. A circular food cup was located at the end of each arm. The light was fixed at 60–65 Lux at the center and 25–30 Lux at the end of each arm. A wide-angle video camera was placed 1.5 m above the center of the maze, allowing real-time monitoring and recording. Animals were food-restricted to keep their body weight at 85% of their initial body weight during the entire duration of the radial-maze experiment to induce a motivational state for food seeking. This food restriction was quite more severe than the one needed in the T-maze because the ERAM test was performed 1 week after the T-maze. This avoided the animals from being habituated to the food restriction. The habituation phase consisted of 3 consecutive days. On the first day, two animals were placed in the maze baited with rewards all along the surface of the eight arms and the cups and were allowed to freely explore during a 15-min session. During the second day, animals were placed alone in the maze baited with rewards in each arm and each cup during a 5-min session. On the third habituation day, rewards were restricted to eight cups, and animals were allowed to freely explore the maze for a maximum of 5 min. The session stopped when the animals consumed all the eight rewards. The training phase consisted of two trials a day for 15 days (5-min session, intertrial interval: 4 h) in which rats were allowed to freely explore the maze and find the rewards in the three baited arms (working and reference memory tasks). Food rewards were placed randomly in three different arms for each animal of the same group and the five other arms were never baited. Between each trial, the maze was cleaned with Ethanol (20%). Each trial was stopped when the three rewards were consumed (maximum duration: 5 min). An arm entry was counted when all four paws entered the arm. The number of reference memory errors (exploring a never-baited arm), working memory errors (exploring an arm previously visited), the total number of errors, and the latency time taken to find the three baits were collected. The training was stopped when the rats fulfilled the following criterion: less than two errors made (total) during three consecutive trials and less than one error made in each trial (20, 22). When animals did not pass the test for any reason (did not look for and/or eat the rewards), the failed trials were not included in the analysis. In this study, the training phase was performed seven weeks after the sequential bilateral transtympanic injection.

### Statistical analysis

Statistical analyses were performed under the supervision of a statistician expert using the GraphPad Prism9 software. Results are expressed as means ± standard error of the mean (SEM). Three-way analysis of variance (ANOVA) and repeated measures (RM-ANOVAs) were used to analyse each parameter of open-field, support surface, and eight-radial arm maze tasks. Two-way ANOVA and repeated measures were used to analyse the scoring and the time course of each symptom. One-way ANOVA (non-repeated measures) was used to analyse Y-maze and T-maze tasks. When ANOVA indicated a significant overall effect, *post hoc* analysis was performed using Tukey's test (after one-way and three-way ANOVA) or Šídák's test (after two-way ANOVA). A significant difference is indicated by ^*^ if *p* < 0.05, ^**^ if *p* < 0.01, ^***^ if *p* < 0.001 and ^****^ if *p* < 0.0001.

## Results

### Assessment of central vestibular compensation

#### Open field and video tracking

**Figure 3:**
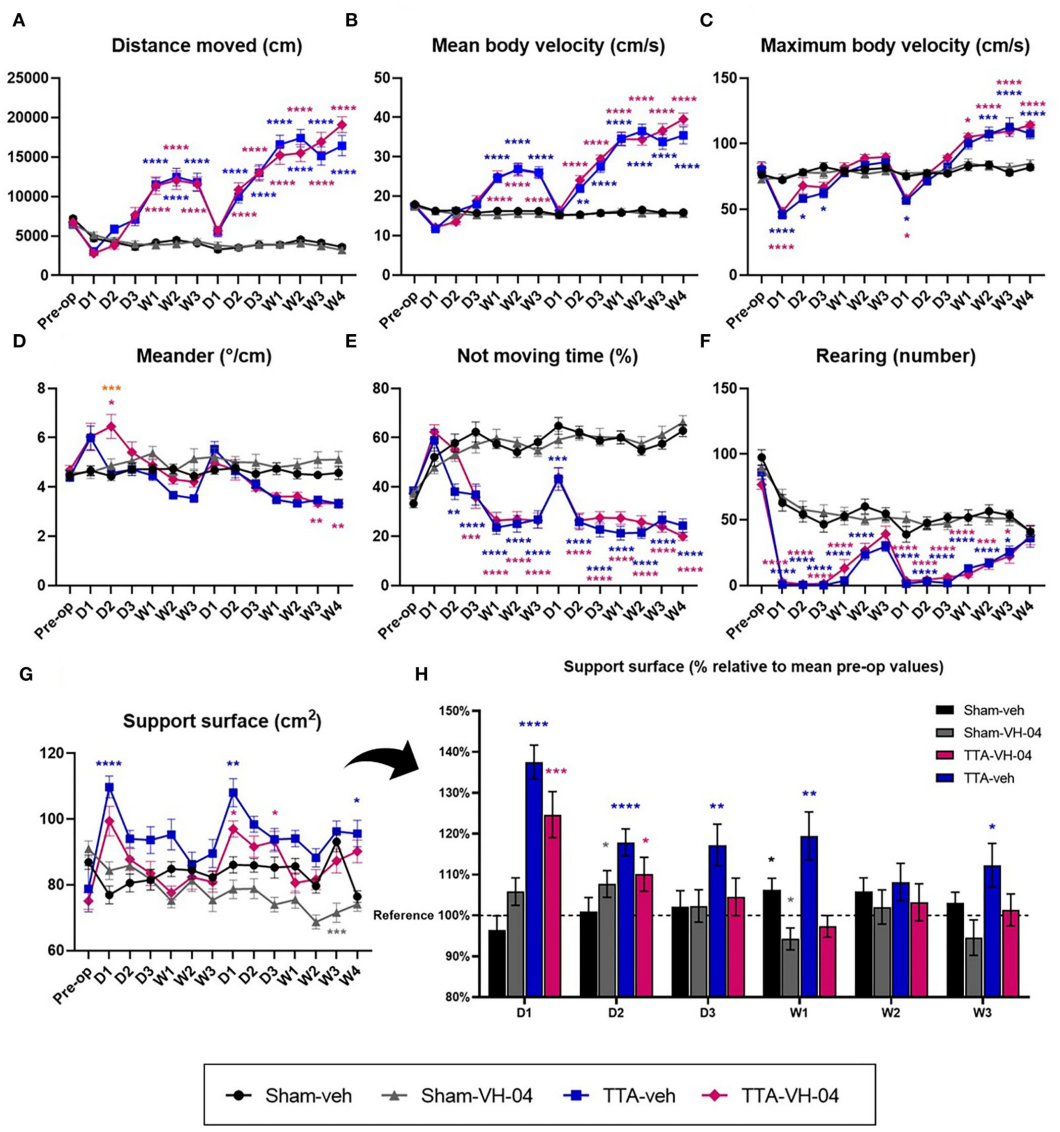
Illustration of the average distance moved **(A)**, mean body velocity **(B)**, maximum body velocity **(C)**, meander **(D)**, not moving time **(E)**, and number of rearing **(F)** automatically measured in a 10 min-session in an open field. Illustration of the support surface area in cm^2^ **(G)** and in % relative to mean pre-op values **(H)**. Data represent mean ± SEM. Three-way ANOVA applies to graphs **(A–G)** and one sample t-test applies to graph **(H)**. **(A–G)** Significant differences between groups Sham-veh and Sham-VH-04, Sham-veh and TTA-veh, Sham-VH-04 and TTA-VH-04, and TTA-VH-04 and TTA-veh are indicated by gray, blue, purple, and orange stars, respectively. **(H)** Significant differences with the mean pre-op value of the four groups (referred to as reference) are indicated by black, gray, blue, and purple stars for Sham-veh, Sham-VH-04, TTA-veh, and TTA-VH-04 groups, respectively. *p < 0.05; **p < 0.01; ***p < 0.001; ****p < 0.0001. Sham-veh and Sham VH-04, *n* = 16; TTA-veh and TTA-VH-04, *n* = 15.

The distance moved is a quantitative parameter that reflects the posturo-locomotor activity of animals and the animal's interest in exploring the open field. Assessment of the distance moved by animals in the open field demonstrated that this parameter did not significantly vary between Sham-veh and Sham-VH-04 groups (Figure 3A). Unilateral and sequential bilateral transtympanic injection of arsanilic acid induced a significant increase in the total distance moved in TTA-VH-04 and TTA-veh groups, relative to their respective pre-op values, between W1 and W3 after the UVL and between D2 and W4 after the sequential BVL (data not shown). These changes displayed statistically significant differences between vestibulo-injured groups and sham groups from W1 to W3 after UVL and from D2 to W4 after BVL (Figure 3A). No statistically significant differences were observed between TTA-VH-04 and TTA-veh groups.

The mean body velocity is a quantitative parameter that reflects the posturo-locomotor activity of animals. Evaluation of the mean body velocity revealed that this parameter did not significantly vary between Sham-veh and Sham-VH-04 groups at any time point (Figure 3B). UVL and BVL produced a significant increase in the mean body velocity in the two TTA groups, relative to their respective pre-op values, between W1 and W3 after UVL and between D2 and W4 after BVL (data not shown). When observed at each timepoint, these changes showed statistically significant differences between vestibulo-injured groups and sham groups between W1 and W3 after UVL and between D2 and W4 after BVL. No statistically significant differences were observed between TTA-VH-04 and TTA-veh groups (Figure 3B).

The maximum body velocity is a quantitative parameter that reflects the posturo-locomotor activity of animals. Unlike the mean body velocity, this is a single value. Evaluation of the maximum body velocity demonstrated that this parameter did not significantly vary between sham groups (Figure 3C). However, this parameter progressed differently from the mean body velocity during central vestibular compensation for vestibulo-lesioned animals (Figure 3C). BVL induced a significant increase in the maximum body velocity in the two TTA groups, relative to their respective pre-op values, between W1 until W4 (data not shown). The maximum body velocity of vestibular-lesioned animals from group TTA-veh significantly decreased at D1 after UVL and then progressively increased from D2 to W1 to finally reach Sham-veh group values (no more significant difference). After BVL, the maximum body velocity of vestibular-lesioned animals from group TTA-veh first decreased, but then increased from D2 to W1 and became significantly different from group Sham-veh from W2 to W4. Between Sham-VH-04 and TTA-VH-04 groups, the decrease was significant at D1 after UVL and BVL and the increase that followed at D2 was significant from W1 to W4 after BVL. Comparison of TTA groups with their respective sham groups revealed a significant benefit of VH-04 over vehicle control at D2 and D3 after UVL. TTA-VH-04 group lost a significant difference vs. Sham-VH-04 group faster relative to TTA-veh vs. Sham-veh groups (Figure 3C).

Meander is defined as “the change in the movement direction of a subject relative to the distance moved and provides an indication of how convoluted the subject's trajectory is”. Sham rats which received VH-04 tended to display a higher deviation of the walk relative to those receiving vehicle control from D2 post UVL, although this tendency was never statistically significant whatever the time point considered (Figure 3D). Following UVL, the mean meander value displayed a strong tendency to increase at D1 and D2 for TTA-VH-04 group and at D1 only for TTA-veh group, although never reaching statistical difference from sham groups except at D2 between Sham-VH-04 and TTA-VH-04 groups. Following BVL, mean meander values for both TTA groups displayed a marked tendency to reduce the deviation of the walking trajectory between W1 and W4, with statistically significant differences relative to sham groups only at W3 and W4 for the TTA-VH-04 group. Surprisingly, a statistically significant difference between the two TTA groups was reached at D2 following UVL. At this specific time point, the walking trajectory was more altered in lesioned rats administered with VH-04 than in rats that received vehicle control.

The not moving time is a quantitative parameter that reflects the posturo-locomotor activity of animals and also the animal's interest in exploring the open field. It can also indicate the stressful state or wellbeing of the animal. Automatised valuation demonstrated that this parameter did not significantly vary between sham groups (Figure 3E). Unlike sham groups, the not moving time of vestibulo-lesioned animals decreased over the days compared to the pre-op values (data not shown). From D2 until W3 after UVL and from D1 until W4 after BVL, the immobility time of animals from the TTA-veh group significantly decreased relative to the Sham-veh group. The same significant difference in the not moving time was observed between Sham-VH-04 and TTA-VH-04 groups, one day later beginning from D3 until W3 after UVL and from D2 until W4 after BVL (Figure 3E). Moreover, no significant difference was observed at each timepoint between the two TTA groups. However, a beneficial effect of Vertigoheel can be observed since the difference compared to their respective sham groups started 1 day after (at D3 for the TTA-VH-04 group instead of D2 for the TTA-veh group after UVL).

Rearing, which is manually calculated, corresponds to the number of times animals stand on their hind paws and reflects the animal's interest in exploring the open field. Assessment of this parameter demonstrated that this parameter did not significantly vary between sham groups (Figure 3F). At each of the 13 time points after pre-op time, the number of rearings of vestibular-lesioned animals was significantly lower relative to their pre-op values (data not shown). The rearing number of animals from the TTA-veh group was significantly different from the Sham-veh group's values from D1 until W2 after UVL and from D1 until W3 after BVL. The same effect was observed between Sham-VH-04 and TTA-VH-04 groups since the rearing number was significantly different between those two groups from D1 to W1 after UVL and from D1 to W3 after BVL. No significant difference was observed between the two TTA groups (Figure 3F).

#### Support surface

The support surface is considered a good estimate of postural control as it reflects the animal's behavioral adaptation compensating for the static vestibulo-spinal deficits induced by the vestibular lesion (25–29). The effects of Vertigoheel vs. vehicle control administration did not statistically differ between sham groups except at W3 after BVL. In vestibulo-injured groups, unilateral and sequential bilateral transtympanic injection of arsanilic acid produced significant enlargement of the support surface area relative to the pre-op values at D1 after UVL for both groups (data not shown). Relative to the corresponding sham group, significant enlargement of the support surface area was reached at D1 after both UVL and BVL and W4 after BVL in the TTA-veh group (Figure 3G). In animals treated with VH-04, the enlargement of the support surface relative to the sham group observed at D1 after both UVL and at D1 and W4 after BVL was significantly less pronounced than for the group injected with vehicle control. However, no significant difference was reached between the two TTA groups. This observation highlighted a significant benefit of Vertigoheel relative to vehicle control at D1 after UVL. To better illustrate the effect of VH-04, each group value was plotted in % relative to pre-op values considered as 100% (mean of the four groups before TT-injection) (Figure 3H). No statistical difference with the pre-op value was observed at D3 until W3 with VH-04 injection whereas the increase of the support surface was still observed at W3 with vehicle control injections (except at W2).

#### Scoring of vestibular syndrome

**Figure 4:**
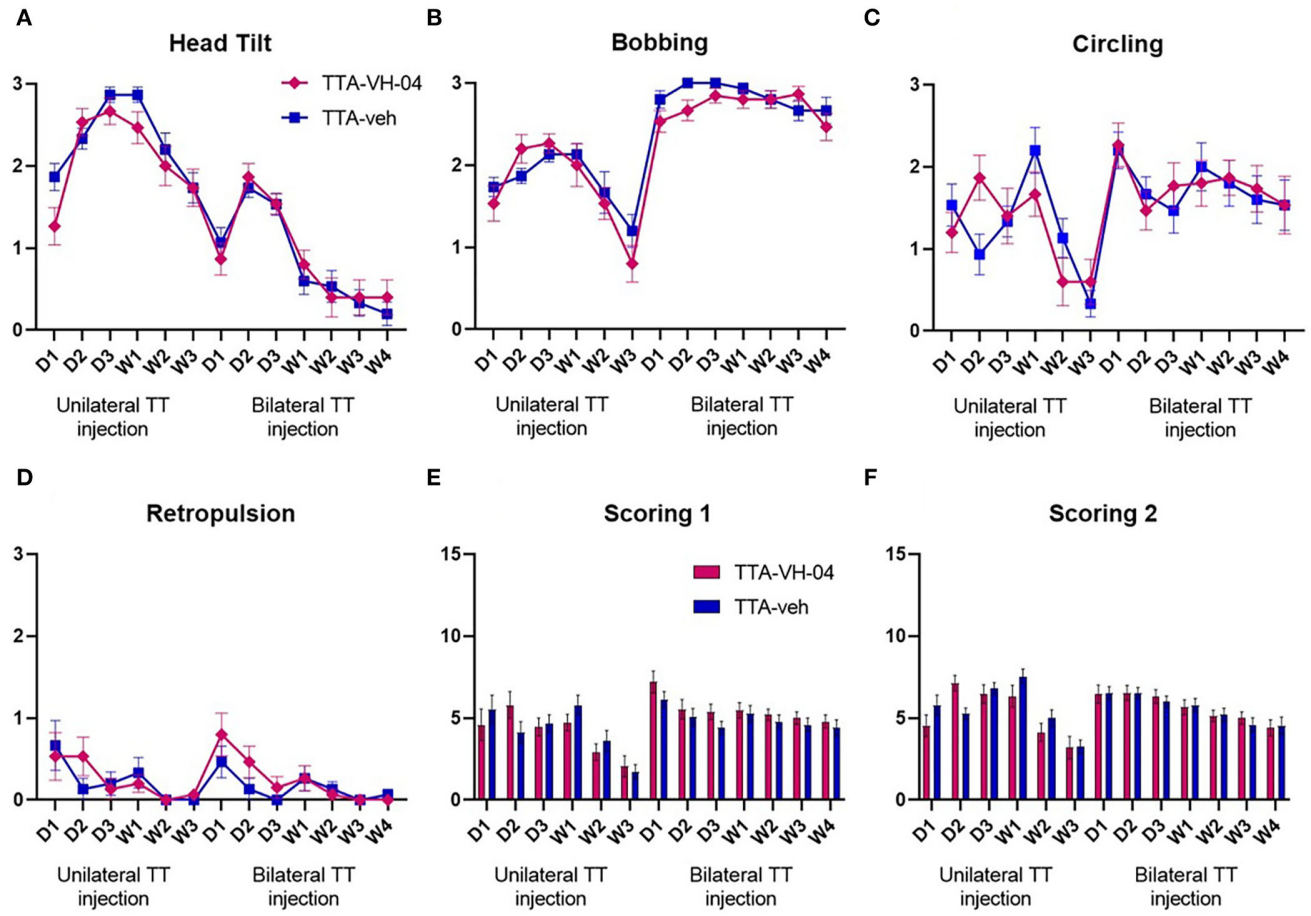
Illustration of the time course of behavioral subjective evaluation score of each symptom: Head tilt **(A)**, Bobbing **(B)**, Circling **(C)**, and Retropulsion **(D)**. Illustration of the time course of behavioral subjective evaluation score involving all symptoms with two different scoring scales **(E,F)**. Data represent mean ± SEM; no statistically significant differences between groups, and two-way ANOVA applies to all graphs. TTA-veh and TTA-VH-04, *n* = 15.

The vestibular syndrome evoked in the rat after vestibular insults are composed of typical symptoms. These symptoms, which include tumbling, retropulsion, circling, bobbing, and head tilt, are together present in the acute phase and sequentially disappear following a specific time course. The vestibular syndrome after BVL displays different kinetics from those in UVL. Some parameters are less prominent, such as circling and retropulsion, while others remain such as alteration of locomotion and pattern of exploration. The severity of the vestibular syndrome displayed by the vestibulo-injured animals was assessed by scoring different parameters [head tilt, bobbing, circling, retropulsion, and tumbling—adapted from (24, 30)] (Figures 4A–D). This evaluation scale is based on the severity of the whole syndrome where five states from the most severe to the least are distinguished (see materials and methods for details). The time course observed in both TTA groups was similar to that published in Lacour et al. (18). The vestibular syndrome was maximum at W1 for the TTA-veh group and D2 for the TTA-VH-04 group after UVL and progressively decreased for both groups until W3 (Figure 4E). After BVL, the syndrome was maximum at D1 for both groups and slightly decreased but remained elevated after 4 weeks. Vertigoheel did not have any effect on the severity of the vestibular syndrome since no significant difference was observed between TTA groups. To be more precise, a different evaluation scale was used. The scoring presented above does not take into account the severity of each symptom itself. In this new evaluation scale, each symptom was assessed from score 0 to score 3. The time course of the vestibular syndrome was the same as the previous scoring. The vestibular syndrome was maximum at W1 for the TTA-veh group and D2 for the TTA-VH-04 group after UVL and progressively decreased until W3 (Figure 4F). After BVL, the syndrome was maximum at D1 for both groups and slightly decreased but remained elevated after 4 weeks. No significant effect of Vertigoheel was observed on the severity of the vestibular syndrome with this new evaluation scale either. The time course of each symptom was also assessed but no significant effect of Vertigoheel was observed in any symptoms (Figures 4A–D). Note that tumbling was not clearly observed in any animals at any time points.

### Assessment of spatial cognition

#### Y-maze

**Figure 5:**
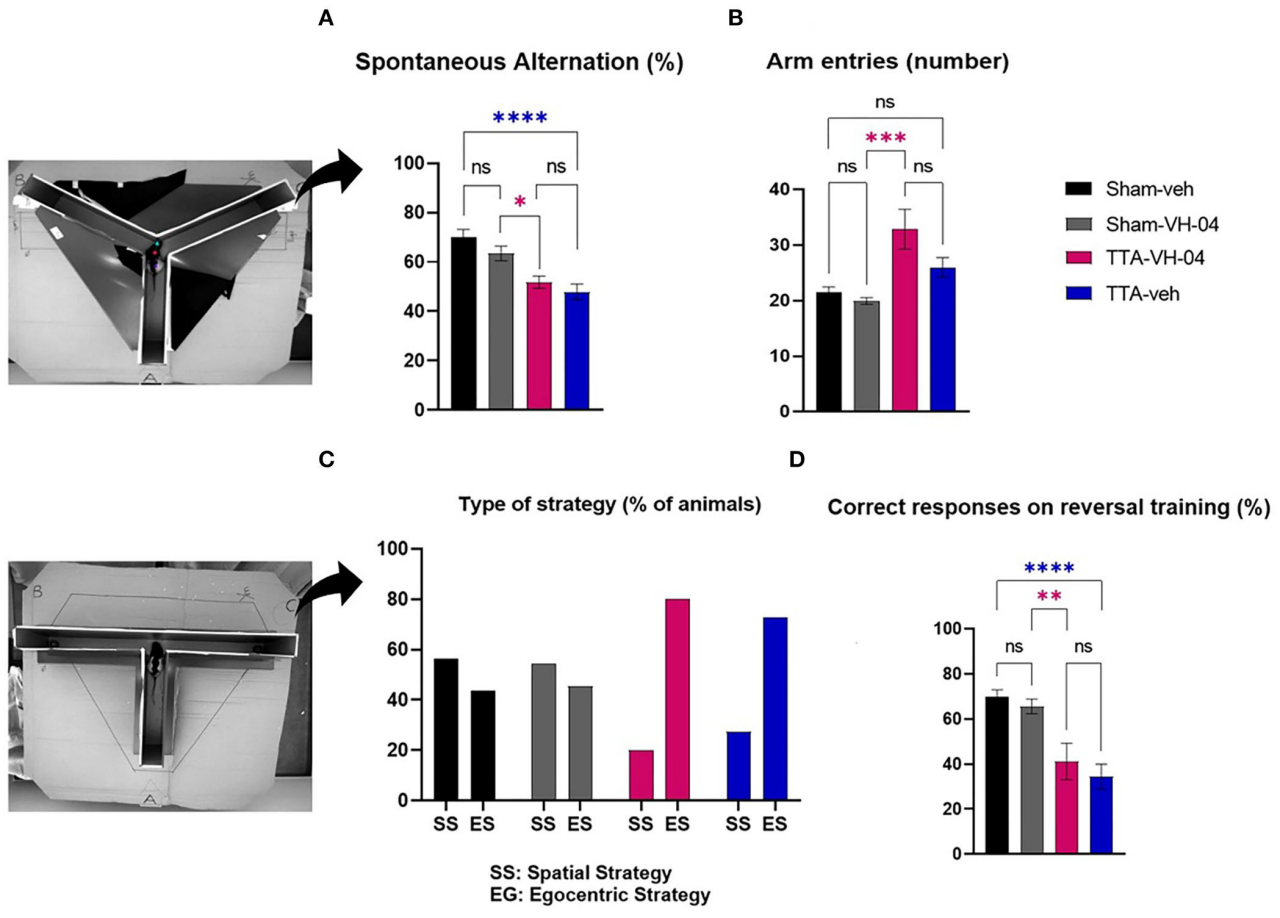
Effect of Vertigoheel on immediate spatial working memory assessed by Y-maze **(A,B)** and on spatial cognitive strategy assessed by T-maze. **(A)** Spontaneous alternation percentage. **(B)** Number of arm entries. **(C)** Type of strategy percentage. **(D)** Percentage of correct responses on reversal training session. Data represent mean ± SEM. One-way ANOVA applies to all graphs except **(C)**. Significant differences between Sham-VH-04 and TTA-VH-04, and Sham-veh and TTA-veh are indicated by purple and blue stars, respectively. *p < 0.05; **p < 0.01; ***p < 0.001; ****p < 0.0001. Sham-veh and Sham VH-04, *n* = 16; TTA-veh and TTA-VH-04, *n* = 15.

The Y-maze evaluates the immediate spatial working memory and is assessed by recording spontaneous alternation in a single session in a Y-maze (31). Spontaneous alternation performances were not significantly different between sham groups (Figure 5A). However, the percentage of spontaneous alternation was significantly different between Sham-veh and TTA-veh groups and between Sham-VH-04 and TTA-VH-04 groups. Vertigoheel did not significantly improve the spontaneous alternation comparing the two TTA groups. The number of arm entries, which indicates the locomotor activity, was significantly altered between the TTA-VH-04 group and the Sham-VH-04 group (Figure 5B). Surprisingly, no significant difference was observed between Sham-veh and TTA-veh groups in the locomotor activity. No significant difference was observed between the two sham groups and between the two TTA groups regarding the number of arm entries.

#### T-maze

The reverse T-maze paradigm tested the learning mechanism and cognitive strategy in finding a food reward. Both TTA and sham groups slowly and gradually located the baited arm during training. The four groups reached the endpoint but on different days: on day 5 for groups Sham-veh, Sham-VH-04, and TTA-VH-04, and on day 6 for group TTA-veh. During the first trial of the reversal training phase, ~56.2% of animals from the Sham-veh group and 54.5% of animals from the Sham-VH-04 group visited the baited arm, indicating a spatial strategy, while the other 43.8 and 45.5%, respectively, visited the non-baited arm, indicating a response strategy (Figure 5C). In contrast, only 20.0% of animals from TTA-VH-04 group and 27.3% of animals from TTA-veh group visited the baited arm indicating that vestibulo-lesioned animals are more likely to use a response strategy (80.0% of animals from TTA-VH-04 group and 72.7% of animals from TTA-veh group chose the non-baited arm). At the end of the reversal phase, the Sham-veh group and Sham-VH-04 group reached 69.8 and 65.6% correct responses, respectively, whereas the BVL group obtained 41.1% correct responses for the TTA-VH-04 group and 34.4% for TTA-veh group (Figure 5D). These differences were statistically significant between Sham-veh and TTA-veh groups and between Sham-VH-04 and TTA-VH-04 groups. No effect of Vertigoheel was observed when comparing the two TTA groups.

#### ERAM

**Figure 6:**
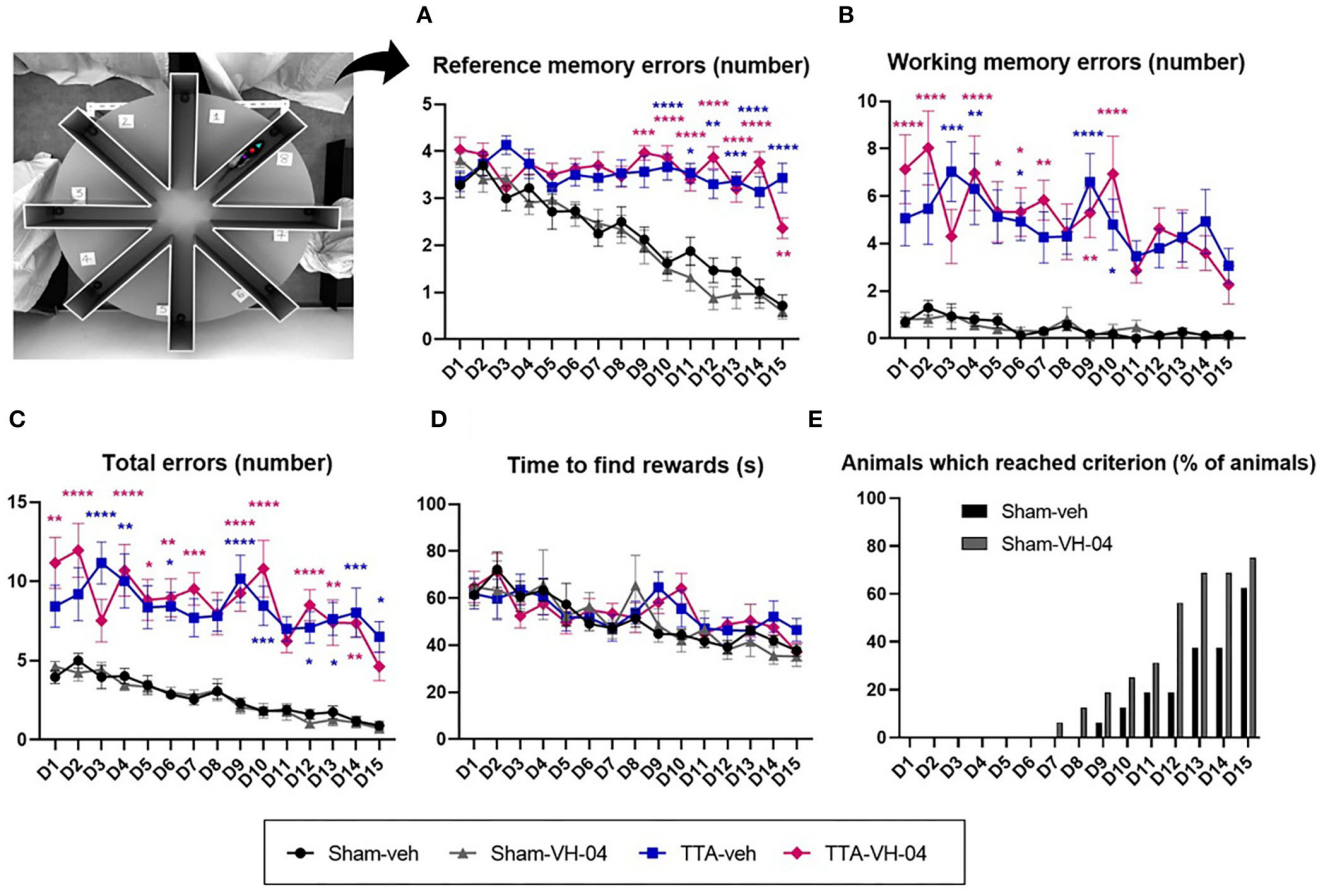
Effect of Vertigoheel on the spatial working and reference memory assessed by the eight radial arms maze. **(A)** Number of reference memory errors: long-term memory. **(B)** Number of working memory errors: short-term memory. **(C)** Total number of errors. **(D)** Mean trial completion time (s). Data represent mean ± SEM. Three-way ANOVA applies to all graphs except **(E)**. Significant differences between Sham-VH-04 and TTA-VH-04, and Sham-veh and TTA-veh are indicated by purple and blue stars, respectively. *p < 0.05; **p < 0.01; ***p < 0.001; ****p < 0.0001. Sham-veh and Sham VH-04, *n* = 16; TTA-veh and TTA-VH-04, *n* = 15.

The ERAM evaluated the spatial working memory and reference memory. No significant difference was observed between the two sham groups or between the two TTA groups in all the parameters observed [reference memory errors (Figure 6A), working memory errors (Figure 6B), total errors (Figure 6C), and time to find the three baits (Figure 6D)]. However, the number of reference memory errors and working memory errors were significantly different between sham groups and TTA groups. Regarding the time to complete the task (consume all three baits), there was no difference between the four groups although the number of entries was increased in the TTA groups. Regarding the percentage of animals that reached the criterion [less than two errors made during three consecutive trials (with one error max. per trial) (32, 33)], it seemed that animals from the Sham-VH-04 group learned slightly faster than animals from the Sham-veh group (Figure 6E). No animals from the TTA groups reached the criterion.

### Thigmotaxis

**Figure 7:**
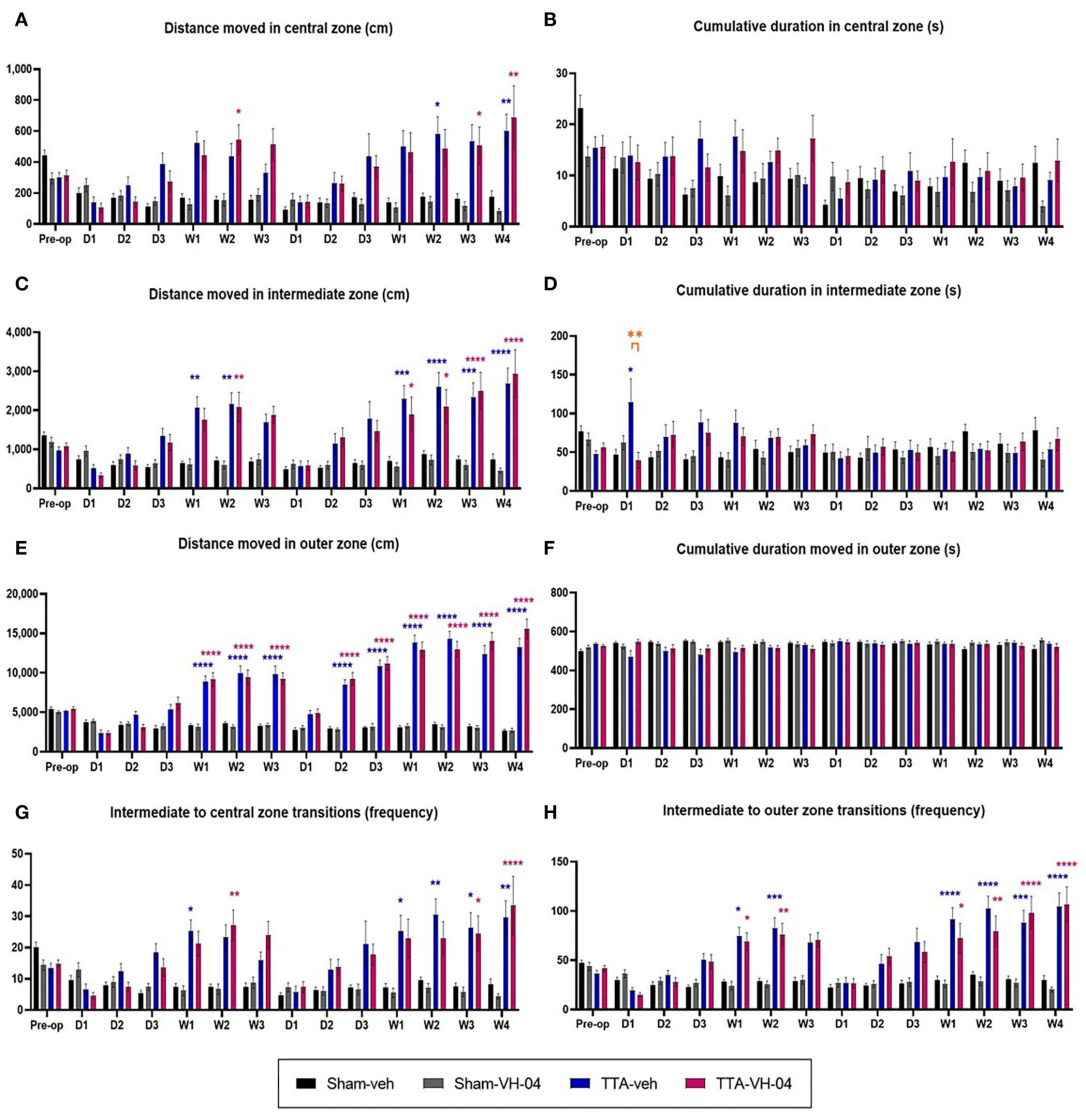
Effect of Vertigoheel on thigmotaxis automatically measured in a 10 min-session in an open field. Illustration of the distance moved **(A)** and the cumulative duration **(B)** in the central zone. Illustration of the distance moved **(C)** and the cumulative duration **(D)** in the intermediate zone. Illustration of the distance moved **(E)** and the cumulative duration **(F)** in the outer zone. Illustration of the number of transitions from intermediate to central **(G)** and intermediate to outer **(H)** zones. Data represent mean ± SEM. Three-way ANOVA applies to all graphs. Significant differences between Sham-VH-04 and TTA-VH-04, Sham-veh and TTA-veh, and TTA-VH-04 and TTA-veh are indicated by purple, blue, and orange stars, respectively. *p < 0.05; **p < 0.01; ***p < 0.001; ****p < 0.0001. Sham-veh and Sham VH-04, *n* = 16; TTA-veh and TTA-VH-04, *n* = 15.

Assessment of the distance moved by animals in the central zone of the open field demonstrated that this parameter did not significantly vary between sham groups (Figure 7A). Vestibulo-lesioned animals from TTA-veh and TTA-VH-04 groups displayed statistically significant differences relative to their respective sham groups at several time points. The distance moved in the central zone by animals from the TTA-veh group was statistically increased at W2 and W4 after BVL relative to the Sham-veh group. Likewise, animals from the TTA-VH-04 group statistically traveled a greater distance in the central zone than animals from the Sham-VH-04 group at W2 after UVL and at W3 and W4 after BVL. No statistically significant differences were observed between the two TTA groups.

Assessment of the cumulative duration in the central zone of the open field demonstrated that this parameter did not significantly vary between sham groups (Figure 7B). Moreover, no statistically significant difference was observed between the Sham-veh group, respectively Sham-VH-04 group, and the TTA-veh group, respectively TTA-VH-04 group. No statistically significant differences were observed between the two TTA groups either.

In the intermediate zone, assessment of the distance moved by animals demonstrated that this parameter did not significantly vary between sham groups (Figure 7C). Unilateral and sequential bilateral TTA produced a significant increase in the distance moved in the intermediate zone in TTA groups, relative to their respective pre-op values (data not shown), which produced statistically significant differences between vestibulo-injured groups and sham groups from W1 to W2 after UVL and from W1 to W4 after BVL (Figure 7C). No statistically significant differences were observed between TTA-veh and TTA-VH-04 groups.

Assessment of the cumulative duration in the intermediate zone of the open field demonstrated that this parameter did not significantly vary between sham groups (Figure 7D). Unilateral and sequential bilateral TTA did not produce any significant difference in the cumulative duration in the intermediate zone between vestibulo-injured groups and sham groups except at D1 after UVL between Sham-veh and TTA-veh groups. At this same time point, D1 after UVL, a statistically significant difference was also observed between the two TTA groups. Note that at this time point, the standard deviation of the TTA-veh group is very high. As is the case in patients, observations in animals show an inter-individual heterogeneity that is classic in the expression of the vertiginous syndrome as in the response to treatments.

The distance moved by animals in the outer zone of the open field did not significantly vary between Sham-veh and Sham-VH-04 groups (Figure 7E). Unilateral and sequential bilateral TTA produced a significant increase in the distance moved in the outer zone in both TTA groups, relative to their respective pre-op values (data not shown). These changes displayed statistically significant differences between vestibulo-injured groups and sham groups from W1 to W3 after UVL and from D2 to W4 after BVL (Figure 7E). No statistically significant differences were observed between TTA groups.

In the outer zone, the cumulative duration did not significantly vary between Sham-veh and Sham-VH-04 groups (Figure 7F). Unilateral and sequential bilateral TTA did not produce any significant difference in the cumulative duration in the outer zone between vestibulo-injured groups and sham groups. No statistically significant differences were observed between TTA-veh and TTA-VH-04 groups.

The number of transitions from the intermediate zone to the central zone did not significantly vary between sham groups either (Figure 7G). Unilateral and sequential bilateral TTA produced a significant increase in the number of transitions between the central and intermediate zones in TTA groups, relative to their respective pre-op values (data not shown), which produced statistically significant differences between vestibulo-injured groups and sham groups at W1 and W2 after UVL and from W1 to W4 after BVL (Figure 7G). The significant differences relative to their sham groups started 2 weeks later for the Vertigoheel group in comparison to the vehicle control group. This delay could be interpreted as a sedative action of Vertigoheel compared to its vehicle. However, this hypothesis is refuted given that there is no statistical difference between TTA-veh and TTA-VH-04 groups.

Assessment of the number of transitions from the intermediate zone to the outer zone demonstrated that this parameter did not significantly vary between the two sham groups (Figure 7H). Unilateral and sequential bilateral TTA produced a significant increase in the number of transitions between the outer and intermediate zones in TTA-veh and TTA-VH-04 groups, relative to their respective pre-op values (data not shown), which produced statistically significant differences between vestibulo-injured groups and sham groups from W1 to W2 after UVL and from W1 to W4 after BVL (Figure 7H). No statistically significant differences were observed between the two TTA groups.

All behavioral assessments and their respective results were summarized in Table 2.

**Table 2:** Summary of the different behavioral assessments and their outcomes.

	**Applied test**	**What has been analyzed?**	**Outcome metric**
Assessment of central vestibular compensation	Open-field test	Behavioral evaluation (distance moved, mean and maximum body velocity, not moving, meander, rearing) to assess posturo-locomotor deficits / dynamic symptoms	Beneficial effect on the alteration of the maximum body velocity and not moving time at day two and three and at day two, respectively, following UVL
		Thigmotaxic behavior: cumulative duration as an index of anxiety and distance moved and the zone transitions as an index of locomotor activity	Vertigoheel does not seem to induce sedative or anxiolytic effects
	Scoring of the vestibular syndrome (severity of the whole syndrome and of each symptom)	Five static or dynamic locomotor deficits (tumbling, retropulsion, circling, bobbing, head tilt)	No effect
	Tail hanging landing test	Support surface to assess postural control/static vestibulospinal deficits	Improvement of postural control at D1 after UVL
Assessment of spatial cognition	Y-maze	Immediate spatial working memory	No effect
	Reverse T-maze	Learning mechanism and cognitive strategy	No effect
	Eight-radial arm maze	Spatial working (short-term) and reference (long-term) memory	Animals from Sham-VH-04 seem to learn slightly faster than animals from Sham-veh group

## Discussion

In this study, we aimed to assess the effect of Vertigoheel compared to the vehicle on central vestibular compensation and cognitive deficits in rats subjected to peripheral vestibular loss. Investigation of central vestibular compensation was made through scoring of the vestibular syndrome, behavior monitored during the open-field test, and the support surface. Investigation of spatial cognition was performed through three sequential behavioral tests: Y-maze, T-maze, and ERAM. Vertigoheel improves central vestibular compensation in rats after an acute UVL, as demonstrated by significant changes relative to placebo on day 1 in postural control, on day 2 in not moving time, and on days 2 and 3 in maximum body velocity. These findings may explain some effects observed in previous clinical studies (12–14) and may contribute to identifying patients with particular vertigo entities benefitting from Vertigoheel.

The literature data on vestibular compensation in animal models are essentially based on a unilateral vestibular injury model. Indeed, this compensation process essentially solicits vestibular information from the intact vestibule. Thus, in this article, the pharmacological effects on vestibular compensation are mainly analyzed and discussed relative to the UVL model.

### The significant beneficial effect of Vertigoheel on the alteration of maximum body velocity and not moving time in vestibulo-injured rats

The significant benefit of Vertigoheel for the recovery of the maximum body velocity and not moving time on days 2 and 3 and on day 2, respectively, following the unilateral insult compared to the other measured parameters (distance moved, mean body velocity, meander, and rearing) demonstrates that the administered compound, at the selected concentration, reached targets involved in the control of these parameters. The lack of effect at D1 after UVL suggests that the Vertigoheel reached functional efficiency only 48 h after the first administration, while repeated administrations allowed significant benefit to be maintained over 48 h. The large heterogeneity in the TTA-VH-04 group may explain the loss of significance between the Sham-VH-04 and TTA-VH-04 groups. Maximum body velocity is a very sensitive parameter that reports the ability of animals to perform very fast displacements. This parameter is one of the most severely affected when the animal displays a dizzy state and/or posturo-locomotor deficits. Improvement of this parameter signals a clear functional benefit of Vertigoheel with regard to the ability of the vestibulo-injured animal to move in its environment. It strongly suggests that the molecular and cellular targets modulated by Vertigoheel may bring a significant reduction of the dizzy state, even if the concentration of Vertigoheel is perhaps insufficient to affect other posturo-locomotor parameters. Based on the beneficial effect of Vertigoheel on the postural stability performed in a situation of syndrome reactivation, monitoring dynamic symptoms in the situation of syndrome reactivation should eventually allow better evidencing of the antivertigo properties of Vertigoheel.

### Lack of benefit of Vertigoheel administration for dynamic vestibular symptoms

The lack of benefit of Vertigoheel for the altered posturo-locomotor parameters (distance moved, mean body velocity, meander, rearing) monitored during the open-field test, may be related to the following issues: the compound may have not reached the molecular effectors involved in the control of the posture and equilibration in the chosen model; this possibility is unlikely as a significant effect of VH-04 has been reported on the postural stability; Vertigoheel was not given at a sufficient concentration to achieve significant benefit; the observed parameters were not performed in the situation of vestibular syndrome reactivation conversely to the analysis of postural stability.

### Lack of statistically significant difference between TTA groups vs. their respective shams at D1 post-UVL in the meander test

As opposed to other parameters, a meander is not sufficiently impacted in vestibulo-injured rats to appear as statistically significant using the three-way ANOVA. Vestibular symptoms in response to unilateral vestibular insults are varied, due to different reflex pathways that originate in the brainstem vestibular nuclei. These symptoms are expressed differentially according to the type of vestibular lesion and at different times after the lesion. This has been well documented previously in vestibulo-injured rats (34) and mice (35). Meander is a highly impacted marker at D1 in the UVN model (36), whereas it is less affected in the transtympanic administration of the kainic acid model (35, 37). In the present study on the TTA model, there is a strong tendency to alter the walking pattern at D1 post-UVL, without being sufficient to achieve a significant difference with sham groups. To our knowledge, meander was not reported as a major marker in the TTA model.

### Reduction of meander in vestibulo-injured rats after BVL

This effect has to be correlated to the increase in the mean and maximum body velocity. In this situation, the rat's walk trajectory is decreased as the animals move faster (38). The observed decrease in meander (degree of walk pattern alteration) after BVL in the two vestibulo-injured groups is stereotypical of better walk control, as animals increase their mean and maximum velocity. This is a particular strategy of rats for avoiding the risk of fall. This can be considered a beneficial strategy.

### Worsening of the walking trajectory (meander) at D2 in UVL rats administered Vertigoheel

No specific disturbance of the TTA-VH-04 group animals on this observational day, which could have explained the observed result, was observed. However, it cannot be ruled out that this effect may be related to an attentional or arousal effect of Vertigoheel leading in turn to an impairment of the walking trajectory. In support of this hypothesis, a higher meander is observed at almost all time points considered in sham animals that received Vertigoheel relative to those which received vehicle control (Figure 3D).

### Administration of Vertigoheel significantly improved postural control at D1 after UVL

Relative to their corresponding sham groups, unilaterally vestibulo-lesioned animals administered Vertigoheel display smaller support surface area than unilaterally vestibulo-lesioned animals administered vehicle control. This is an interesting result as it clearly shows that Vertigoheel significantly improves the postural stability in vestibulo-injured animals. Although this effect is only statistically significant at 24 h after the vestibular insult, the effect of Vertigoheel is maintained until 3 weeks after UVL. Conversely, vestibulo-lesioned animals injected with vehicle control display a tendency of enlarged support surface until W1 after UVL. At the acute delay D1, there is no statistically significant difference in the Vertigoheel vestibulo-lesioned group compared to the Vertigoheel sham group using the three-way analysis of variance. This is particularly relevant when considering that without treatment, this postural parameter is significantly enlarged until one month in the same unilaterally rodent arsanilic acid model (39). This indicates that Vertigoheel, at the concentration used, is able to induce immediate and persistent functional changes that impact the postural stability of the animals. This also suggests that the cellular and molecular mechanisms impacted by Vertigoheel are expressed within a short time window following the vestibular insults. It remains unclear why the effect of Vertigoheel is significant in the study of postural stability, while no benefit could be observed with regard to the dynamic parameters mentioned above (distance moved, mean body velocity, etc.). This difference is likely to be based on the method of quantifying the support surface. This postural parameter is measured in the situation of reactivation of the vestibular syndrome by the tail hanging landing method (30). This method makes it possible to rule out the other sensory modalities involved in central vestibular compensation (tactile and proprioceptive information from the pads of the four paws of the animal, information from the visual system). It also accentuates the electrophysiological asymmetry between the two homologous vestibular nuclei by subjecting the animal to a vertical linear acceleration (30). In the acute phase, the peripheral deafferentation causes an electrophysiological asymmetry: the injected side becomes silent (no more activity in the vestibular nuclei), while the healthy side remains intact (normal electrical activity). This electrophysiological asymmetry is responsible for the observed vestibular syndrome. Central vestibular compensation is carried out both by endogenous (modulation of cell membrane effectors and neurotrophins) and exogenous (proprioceptive, tactile, and visual inputs, together with motor information) mechanisms. Upon syndrome reactivation (vertical lift) condition, the exogenous inputs are significantly reduced allowing partial removal of central vestibular compensation. The measured syndrome is higher at D1 than in the following days because the endogenous mechanisms become progressively more efficient over days and weeks after the lesion. Given the curve of the TTA-VH-04 group (which is similar to that of the Sham-VH-04 group; Figure 3G), it might be suggested that the compound stimulates the expression of these endogenous mechanisms in the VNs disconnected from peripheral sensors. Despite the exacerbation of the syndrome, Vertigoheel seems to improve postural stability. These pharmacological effects may be similar to findings from previous work on the feline UVL model on the same postural parameter with betahistine (27, 29) and tanganil (28), two anti-vertiginous compounds of reference in human clinical practice (40). Electrophysiological homeostasis of vestibular nuclei is considered to be the key parameter of central vestibular compensation (2). Vertigoheel may be a modulator of neuronal excitability. This hypothesis is supported by the demonstration that Vertigoheel acts specifically when the syndrome is reactivated. Another possibility would be that this compound induces a pro-cognitive action (41–43). It is known that central vestibular compensation is considered a process of sensory-motor relearning involving structures, such as the hippocampus. Like the hippocampus, neuroplasticity mechanisms such as LTP, LTD, and neurogenesis have also been demonstrated in vestibular nuclei (36, 44). It is possible that Vertigoheel targets such mechanisms and promotes postural recovery. Vertigoheel may also reduce the postural stability reaction (area between the four paws) following reactivation of the syndrome *via* an anxiety-relieving action.

Monitoring dynamic symptoms in a situation of syndrome reactivation should allow better evidence for the antivertigo properties of Vertigoheel. We recently reported a study using the Dynamic Weigh Bearing device to finely monitor the changes in weight distribution and the center of gravity over the first days following the vestibular insult (45). Such an approach should enable better appreciation of changes in the weight distribution that takes place subsequently to loss of vestibular inputs as well as the effect of Vertigoheel on these parameters.

It would also be interesting to identify neurobiological correlates supporting the pharmacological effects of Vertigoheel (e.g., highlighting the effects of the product on neurogenesis, neuronal excitability, inflammation, stress, etc.) using immunohistochemical approaches (such as those described below).

### No significant effect of Vertigoheel on the vestibular syndrome severity

As with the analysis of dynamic symptoms, the subjective analysis of vestibular syndrome was performed in a spontaneous, non-reactivated condition. This may explain the lack of effect of Vertigoheel. It might be interesting to carry out these qualitative measures after reactivation of the syndrome, as detailed above.

### Spontaneous alternation and locomotor activity in the Y-maze

The results of the Y-maze are similar to those from our previous study (20). Both TTA-veh and TTA-VH-04 groups displayed a similar decrease of spontaneous alternation compared to Sham-veh and Sham-VH-04, respectively. They also revealed impairment of spatial function related to Y-maze alternation following BVL without any drug effect. Vertigoheel has an impact neither on the immediate spatial working memory nor on the locomotor activity. The spontaneous alternation is normal in sham groups (70 and 60%) and near random (50%) in TTA groups meaning that the test was properly performed with a significant difference.

The number of entries measures the locomotor activity more than a spatial cognitive aspect. To our surprise, we did not observe a significant difference between Sham-veh and TTA-veh groups as expected as a result of the hyper-locomotor activity and spatial troubles, although we observed a significant difference between the Sham-VH-04 group and TTA-VH-04 group. The lack of difference in sham conditions may tentatively be explained by a group effect. Additional experiments will be required to ascertain the drug effect.

### Ego vs. allocentric strategy in the reverse T-maze

Sham groups use both egocentric and spatial strategies indifferently, as reported in the literature including our previous data (21). There does not appear to be an effect of Vertigoheel on this parameter. The vestibulo-injured groups preferentially use egocentric strategy, i.e., somesthesia-centered since vestibular information is absent and likely impairs visuo-vestibular integration within the hippocampus and thus spatial memory (46–48). Vertigoheel does not improve the spatial memory-based strategy.

### Learning and spatial memory in the eight-radial arms maze

Both sham groups show a good learning curve with a speed of task completion comparable to the literature (20, 22). Conversely, the vestibulo-injured groups have poor spatial memory performances with a still high number of reference memory errors. However, the rewards are found within the same time as the sham groups, as reported in a previous study (20), demonstrating that the TTA groups understood the task well, performed it very quickly but randomly without any spatial learning, and compensate with a strategy of increasing their locomotor activity, but chose arms randomly [Figure 6D is a graph illustrating this point and similar to our previous studies (20, 22)]. Moreover, the working memory remains partly or weakly impaired since it does not involve vestibular inputs directly and shows the specificity of the test to discriminate spatial memory impairments. VH-04 compound did not improve spatial memory performances.

### Effect of Vertigoheel in stimulating the learning phase

As illustrated in Figure 6E, the animals from the Sham-VH-04 group seem to learn slightly faster than animals from the Sham-veh group. Although it cannot be ruled out that this may reflect a group discrepancy in the ability to learn the position of the three rewards, this could also be due to a pharmacological effect of Vertigoheel.

### Thigmotaxis-effect of Vertigoheel on the distance moved in the three zones

Thigmotaxis is a phenomenon according to which anxious animals tend to spontaneously explore areas close to the walls, whereas relaxed animals will rather explore the inner area (49). As the thigmotaxic behavior is being considered a symptom of anxiety, we quantified this parameter in vestibulo-injured animals and verified whether it was impacted by Vertigoheel or its vehicle.

Patients who suffer from a vestibular deficiency or dysfunction present a vestibular syndrome which is associated with anxiety symptoms (50, 51). These anxiety symptoms indicate that vestibular function and emotional processes interact together (23). Anxiety-like behavior in rodents can be explored through different behavioral tests, such as the elevated plus maze (52), the black and white box test (22), and the open-field test (53). In this new study, we have focused on the latest test, i.e., the open-field test, to explore the thigmotaxic behavior.

The results of this study replicate data from the UVN rodent model (38) and the bilateral vestibular deafferentation rodent model with arsanilic acid (53). The UVL, whether induced by arsanilic acid or by a section of the vestibular nerve, leads to an increase in exploration behavior that results in a significant increase in the distance moved in the open field. The BVL with arsanilic acid further alters this parameter, the distance moved is significantly higher compared to that observed in unilaterally injured animals [present study, (53)].

Generally speaking, when the distance moved is analyzed in the four different groups, we notice that whatever the group analyzed, the animals preferentially moved to the zones on the edge of the open field. This is true for both sham groups and can be interpreted as an anxiety behavior based on the definition of thigmotaxis. Despite the habituation period to the open field whose main objective is to limit stress, animals may still be more or less stressed by the environment (device, experimenter, etc.). Similarly, our results show that vestibulo-injured rats in TTA-veh and TTA-VH-04 groups have a significantly higher distance moved than sham groups and preferably in areas bordering the open field (outer zone).

**Figure 8:**
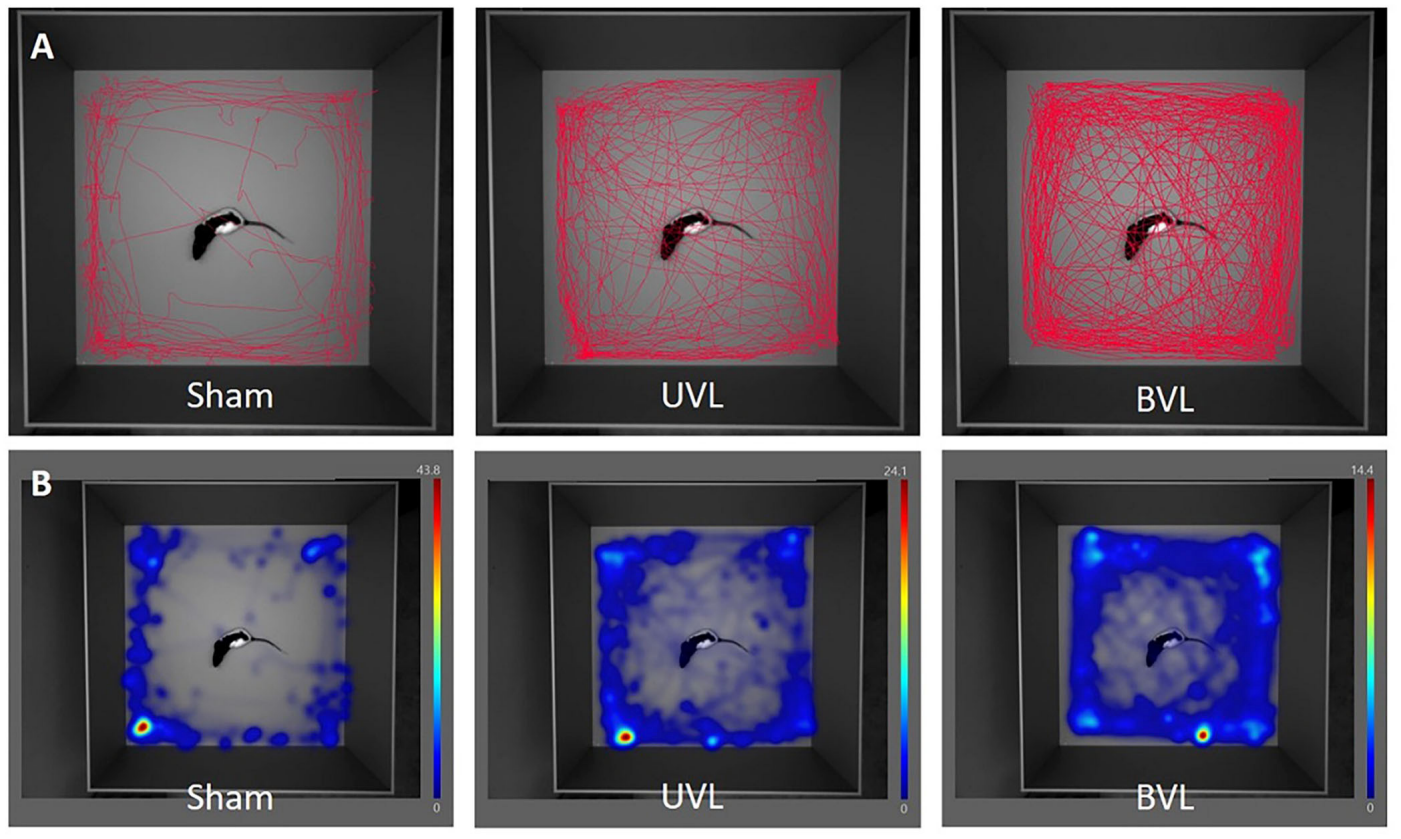
Movement tracing **(A)** and heatmaps **(B)** of center-point over 10 min analysis in the open-field at W3 after sham surgery (left), at W3 after unilateral TT injection (middle), and at W4 after the sequential bilateral TT-injection (right).

After UVL, animals from TTA groups tended to explore the central zone more than animals from sham groups even if the only significant difference was at W2 between Sham-VH-04 and TTA-VH-04 groups. After BVL, the difference was significant at W2, W3, and W4, suggesting that BVL animals further explored the central zone compared to sham groups. Nevertheless, this increase in the distance moved is even more evident in the intermediate zone and the outer zone. The difference between TTA groups and their relative sham groups is statistically significant at W2 and from W1 to W2 in the intermediate zone after UVL for TTA-VH-04 and TTA-veh groups, respectively, and from W1 to W4 in the intermediate zone after BVL for both groups. In the outer zone, the distance moved is statistically different from W1 to W3 after UVL and from D2 to W4 after BVL for both TTA groups. Thus, the longer distance traversed in the central zone by vestibulo-injured animals compared to their respective sham groups seems to be due to an increase in their locomotor activity following BVL and not be related to decreased anxiety since it was also observed in the intermediate zone and even more in the outer zone. This hyperactivity has already been shown in several studies (54–58) such as in the present study on Vertigoheel in which TTA animals displayed a significant increase in the total distance moved in the open field (Figure 8). The reason why BVL displays a higher level of locomotor activity remains unclear. A recent study using the UVL model in rodents shows a reconfiguration of the cerebral connectome (59). A mosaic of brain structures including the motor cortex in both hemispheres recalibrates to promote central vestibular compensation. The emergence of this new motor network may partly explain the increase in locomotor activity observed in vestibulo-lesioned rats. Thus, it is possible that the attendance in the peripheral zone of the open field increases because the vestibulo-lesioned animals display a higher exploratory activity generally exacerbated in all zones of the open field. It can also be postulated that thigmotaxic behavior is the result of anxiety generated by the vestibular syndrome. To refute or confirm these hypotheses, it would be interesting to treat animals from both TTA groups with an anxiolytic compound to see if the thigmotaxic parameter is affected. Moreover, because the cumulative duration parameter reflecting the thigmotaxic behavior as an index of anxiety was not affected between TTA and sham groups, the increase observed in the distance moved and the zone transitions are more likely to be caused by an increase in the locomotor activity following the vestibular insult.

Among the three measured parameters, the distance moved (and the zone transitions) rather reflects the thigmotaxic behavior as an index of locomotor activity. In the present study, no statistically significant difference in the distance moved parameter was observed between the two sham groups or between the two TTA groups. This lack of significant differences between vehicle control and Vertigoheel groups reveals that Vertigoheel does not seem to induce a sedative effect. It can indeed be argued that if Vertigoheel induced a sedative effect, the distance moved would be reduced in Sham-VH-04 and TTA-VH-04 groups compared to Sham-veh and TTA-veh groups, respectively. This non-sedative effect can be beneficial (at least non-deleterious) for vestibular and memory function. It is generally accepted in the literature that sedative drugs are considered “vestibulodepressant or vestibuloplegic” compounds as they delay the development of central vestibular compensation [(60) for review]. Similarly, a recent study undertaken by Vertidiag on the same model shows that treatment with fluoxetine, an antidepressant compound, aggravates vestibular deficits (61). Fluoxetine is probably effective in reducing the stress and anxiety generated by vestibular lesion but is not beneficial for balance function recovery. It is also interesting to note that anxiolytic medications from the benzodiazepines family can affect memory, usually by causing sedation or confusion (40, 62). However, full validation of this idea would require further studies including better-suited behavioral tests that would accurately quantify anxiety and the use of specific histological markers of stress and anxiety. Concerning memory function, central vestibular compensation is considered by some authors as sensorimotor relearning (after a vestibular insult one relearns to walk and to stand in balance). These processes involve hippocampus intervention. In general, all compounds that promote memory can be beneficial for central vestibular compensation. Despite inconclusive results on the memory function in the present study on Vertigoheel, an effect on memory function cannot be categorically ruled out since too many parameters have to be taken into account (dosage of the study medication, optimal therapeutic window, etc.). In the same way, regarding excitability, it would be interesting to verify whether Vertigoheel modulates the level of excitability of cerebral structures, such as the vestibular nuclei. Compounds that facilitate neuronal excitability are believed to be beneficial for central vestibular compensation (63). This work could be carried out using specific histological markers of neuronal excitability (KCC2, SK, GABAa, etc.).

### Effect of Vertigoheel on the cumulative duration in the three zones

The cumulative duration parameter rather reflects the thigmotaxic behavior as an index of anxiety. Except at D1 after UVL in the intermediate zone, assessment of the parameter “cumulative duration” in the different zones did not reveal any statistically significant difference between each group at every time point. This significant difference at D1 between TTA-veh and TTA-VH-04 groups and Sham-veh and TTA-veh groups might be explained by the heterogeneity of animals from the TTA-veh group. This lack of difference can be explained by the hyperactivity of vestibulo-lesioned animals. BVL animals do not often stop and are constantly moving. Conversely, sham animals stop much more often (for example for grooming) and have a rearing number that is statistically increased compared to lesioned animals. This might explain why we did not see a difference in the cumulative duration between the 4 groups in each zone whereas we can observe it with the distance moved.

Conversely to the distance moved and the transitions number parameters, the lack of any statistical difference between the two sham groups and the TTA groups on the cumulative duration in any of the three zones suggests that the vestibular lesion did not have any effect on this parameter.

Given the absence of a significant difference in the cumulative parameter between sham groups, it might be suggested that Vertigoheel at this concentration and in this experimental protocol does not affect thigmotaxic behavior. It could therefore be postulated that Vertigoheel does not have anxiolytic properties in this paradigm. This result would be of interest and should be taken into account with regard to the anxiolytic (and sedative) actions of some pharmacological compounds on central vestibular compensation and memory function (40, 64). To confirm this hypothesis, more suitable tests such as the black and white box test could be explored. In the study by Machado et al. (21), BVL animals that received chronic treatment of diazepam displayed reduced anxiety compared to BVL control animals (more time spent in the white box). However, an effect of Vertigoheel on anxiety cannot be definitively ruled out and further study including positive control (a group administered with an anxiolytic compound) is needed to conclude the effect of Vertigoheel on thigmotaxis.

### Effect of Vertigoheel on the transition between zones

The number of transitions from the intermediate zone to the outer zone was significantly increased at W1 and W2 after UVL and from W1 to W4 after BVL for both TTA groups. Similarly, this increase in the number of transitions was also statistically significant from the intermediate zone to the central zone at W1 after UVL and from W1 to W4 after BVL for the TTA-veh group and at W2 after UVL and at W3 and W4 after BVL for TTA-VH-04 group. This increase in the number of transitions in all zones might also be explained by the higher level of locomotor activity of vestibulo-injured animals from both TTA groups. Moreover, even if these groups seemed to perform far more transitions between the intermediate and the central zones (between 20 and 30 transitions for animals from TTA groups vs. about 10 transitions for animals from sham groups), this number remains 2–3 times below the number of transitions they made between the intermediate and the outer zones (between 50 and 100 transitions for animals from TTA groups vs. <50 transitions for animals from sham groups).

### Limitations of the study

One limitation of this study is that it was performed on only male rats. However, considering the translational context of this research and the fact that females are more likely to display vestibular pathology than males, the next step should be to investigate the effect of Vertigoheel administration in female vestibular-injured rats. The behavioral evaluation of the different rat groups has not been performed with randomization between groups nor blindly. This could have brought potential sources of bias to the study.

## Conclusion

This study reveals the significant benefit of Vertigoheel on central vestibular compensation following a unilateral peripheral vestibular loss, as demonstrated by improvement of specific symptoms. This result is of interest with a view to developing pharmacological strategies to alleviate vestibular disorder symptoms in humans. Indeed, there is currently a strong medical need for efficient medication for peripheral vestibulopathies and there is limited expert consensus on treatment recommendations so far. However, further investigations will be necessary to identify the best conditions to achieve the optimum benefit of Vertigoheel to be used as an antivertigo drug.

## Data availability statement

The original contributions presented in the study are included in the article/supplementary material, further inquiries can be directed to the corresponding author/s.

## Ethics statement

The animal study was reviewed and approved by Ethics Committee no. 036 from the French National Committee of Animal Experimentation.

## Author contributions

AM, BS, CC, and BT conceived the study. BH and RB performed behavioral pharmacology and performed the TTA injections. BH, RB, NC, CC, and BT contributed to the data analysis and interpretation of the results. BH, CC, and BT wrote the manuscript. BH, RB, CB, NC, SB, AM, KW, BS, BT, and CC contributed to its editing. All authors contributed to the article and approved the submitted version.

## Funding

This study was funded by Heel GmbH.

## Conflict of interest

Authors BH, RB, CB, and NC were employed by company Vertidiag. Authors AM, KW, and BS were employed by Heel GmbH. The authors declare that this study received funding from Heel GmbH. The funder had the following involvement: study design.

## Publisher's note

All claims expressed in this article are solely those of the authors and do not necessarily represent those of their affiliated organizations, or those of the publisher, the editors and the reviewers. Any product that may be evaluated in this article, or claim that may be made by its manufacturer, is not guaranteed or endorsed by the publisher.
